# An advanced method for propargylcholine phospholipid detection by direct-infusion MS

**DOI:** 10.1016/j.jlr.2021.100022

**Published:** 2021-01-13

**Authors:** Mohamed H. Yaghmour, Christoph Thiele, Lars Kuerschner

**Affiliations:** LIMES Life and Medical Sciences Institute, University of Bonn, Bonn, Germany

**Keywords:** click, lipidomics, lysophosphatidylcholine, ether lipid, plasmalogen, propargyl-PC, AcCN, acetonitrile, LpPC, lyso-propargyl phosphatidylcholine, LpPC O, ether-linked lyso-propargyl phosphatidylcholine, NL, neutral loss, N3Pal, azidopalmitate, PC, phosphatidylcholine, PC O, ether phosphatidylcholine, PE, phosphatidylethanolamine, pPC, propargyl phosphatidylcholine, pPC O, ether-linked propargyl phosphatidylcholine, PS, phosphatidylserine, pSM, propargyl sphingomyelin, SM, sphingomyelin, TG, triglyceride

## Abstract

Phospholipids with a choline head group are an abundant component of cellular membranes and are involved in many important biological functions. For studies on the cell biology and metabolism of these lipids, traceable analogues where propargylcholine replaces the choline head group have proven useful. We present a novel method to analyze propargylcholine phospholipids by MS. The routine employs 1-radyl-2-lyso-sn-glycero-3-phosphopropargylcholines as labeled lysophosphatidylcholine precursors, which upon cellular conversion direct the traceable tag with superb specificity and efficiency to the primary target lipid class. Using azidopalmitate as a click-chemistry reporter, we introduce a highly specific, sensitive, and robust MS detection procedure for the propargylcholine phospholipids. In a first study, we apply the new technique to investigate choline phospholipid metabolism in brain endothelial cells. These experiments reveal differences in the metabolism of phosphatidylcholine and its pendant, ether phosphatidylcholine. The novel method described here opens a new, quantitative, and detailed view on propargylcholine phospholipid metabolism and will greatly facilitate future studies on choline phospholipid metabolism.

Phospholipids containing a choline moiety in their head group represent a major fraction of the cellular lipidome. The family of choline phospholipids includes phosphatidylcholine (PC), ether phosphatidylcholine (PC O), and sphingomyelin (SM). In most eukaryotic cells, PC comprises almost half of all phospholipids, whereas the generally less abundant SM or PC O show highly elevated levels in particular cells, e.g., in the brain or the heart.

For investigations on the cell biology of choline phospholipids, an analogue of choline, propargylcholine, bearing a terminal alkyne moiety, was introduced (1). Upon metabolic incorporation, the propargylcholine replaced the majority of choline head groups in the cellular lipidome. The terminal alkyne of the propargylcholine phospholipids can be click reacted (2) with dedicated reporter azides (3) to enable lipid tracing by microscopy (1).

A particular strength of this tracer is the fact that the obtained localization data can be correlated with metabolic analyses. Propargylcholine phospholipid metabolism can be followed by TLC using fluorogenic reporter azides (4, 5) or by MS benefiting from a specific precursor ion in positive ion mode (1). However, both technologies have major limitations. For TLC, the limit of detection is in the low picomole range, and usually, lipid species are not resolved (4). While MS considerable boosts sensitivity, the conventional approach to detect the main propargylcholine lipid metabolites only delivers their sum FA composition (1).

We have recently introduced a highly sensitive MS method for tracing alkyne-labeled lipids employing a dedicated azide reporter that upon click reaction facilitates the ionization and identification of the labeled product (6). Using this reporter, termed C171, we demonstrated subfemtomole sensitivity for side chain-labeled alkyne-lipid tracing.

Here, we demonstrate the applicability of our C171-based method for analyzing also head group-labeled alkyne lipids, the propargyl phospholipids. As the different positioning of the alkyne label at the head group imposes some intrinsic restrictions, we furthermore present a novel method overcoming these limitations. We therefore introduce azidopalmitate (N_3_Pal) as a clickable MS reporter that allows for direct identification of the labeled propargyl phospholipids at the MS1 level by conferring a predictable mass shift to the analyte. Importantly, in negative ion mode at the MS2 level, a diagnostic fragment is formed that confirms the lipid identity while the individual side chains of the lipid are revealed in parallel. We have used this novel method in a series of experiments where we investigated the propargyl phospholipid metabolism in a brain endothelial cell line. This study opens a detailed and quantitative view on phosphocholine lipid homeostasis in bEND3 cells and demonstrates differences between regular and ether PC metabolism.

## Materials and Methods

### Lipid and chemical probes

Palmitoyl-lyso-propargyl-PC (LpPC 16:0; supplemental Fig. S1A) was synthesized as before (5). The ether analogue 1-O-hexadecyl-2-lyso-sn-glycero-3-phosphopropargylcholine (LpPC O-16:0; supplemental Fig. S1B) was synthesized analogously: 1-O-hexadecyl-2-oleoyl-sn-glycero-3-phosphocholine (Avanti; 878112) was dissolved in diethylether and combined with a solution of propargylcholine bromide (Sigma; P51001) and phospholipase D (*Streptomyces* spec.; Sigma; P4912) in acetate buffer (100 mM sodium acetate, pH 5.6, 40 mM CaCl_2_). After vigorous stirring at 30°C for 24 h, the organic phase was separated, and the solvent evaporated. The residue was separated by silica column chromatography (CHCl_3_/methanol/water 65/25/2) to yield 1-O-hexadecyl-2-oleoyl-sn-glycero-3-phosphocholine, propargyl phosphatidylcholine (pPC) O-16:0/18:1. *Crotalus atrox* snake venom was dissolved in borate buffer (100 mM boric acid, pH 7.1, 20 mM CaCl_2_) and combined with a solution of pPC O-16:0/18:1 in diethylether/methanol 49/1. After vigorous stirring at 30°C for 4 h, the solvents were evaporated. The residue was separated by silica column chromatography (CHCl_3_/methanol/water 65/35/8) to yield LpPC O-16:0. The synthetic pPC 31:1 used as internal standard for MS analysis was synthesized from PC 31:1 (7) analogously to the method described for pPC O-16:0/18:1. Alike, the synthetic pPC 18:0/18:0, pPC 18:1/18:1, pPC 18:2/18:2, pPC 18:3/18:3, pPC 20:4/20:4, and pPC 22:6/22:6 used for testing the performance of the method were synthesized from PC 18:0/18:0 (Avanti; 850365), PC 18:1/18:1 (Avanti; 850375), PC 18:2/18:2 (Avanti; 850385), PC 18:3/18:3 (Avanti; 850395), PC 20:4/20:4 (Avanti; 850397), and PC 22:6/22:6 (Avanti; 850400), respectively.

The MS-reporter azide (N_3_Pal) was synthesized as follows. 16-Bromohexadecanoic acid (Sigma; 568708) was stirred with sodium azide (Sigma; 8223350) in dimethyl sulfoxide for 24 h. Hexane/ethylacetate 3/1 was added to the reaction mix before several extractions with water. The organic phase was separated, and the solvent evaporated. Pure N_3_Pal was crystallized from hexane/ethylacetate 3/1. The MS-reporter azide (C171) has recently been described (6).

### Cell culture and lipid labeling

The brain endothelial cell line bEND3 was obtained from ATCC (CRL-2299) and maintained in DMEM medium (Gibco; 31966021) containing 10% fetal calf serum (Gibco; 11560636) and 1% penicillin/streptomycin (Gibco; 15070063). Propargylcholine lipids were added to the medium at concentrations of 20 μM from 5 to 10 mM stock solutions in 80% ethanol. Cells were then cultured for 24 h.

### Lipid extraction and click reaction

Cells on 24-well dishes (supplemental Fig. S2) were washed once with ice-cold PBS (Sigma; 806552) and quickly once with 155 mM ammonium acetate, taking care to remove the liquid after the last wash as completely as possible. The lipids were extracted by addition of 500 μL methanol:CHCl_3_ 5/1 containing 240 pmol pPC 31:1, 210 pmol phosphatidylethanolamine (PE) 31:1, 396 pmol PC 31:1, 98 pmol phosphatidylserine (PS) 31:1, 56 pmol phosphatidic acid 31:1, 51 pmol phosphatidylglycerol 28:0, 39 pmol lysophosphatidate 17:0, 35 pmol lysophosphatidylcholine; 17:1, 38 pmol lysophosphatidylethanolamine (LPE) 17:1, 32 pmol ceramide 17:0, 99 pmol SM 17:0, 55 pmol glucosylceramide 12:0, 339.7 pmol triglyceride (TG) 50:1, 111 pmol cholesteryl ester 17:1, 64 pmol diglyceride 31:1, and 103 pmol monoglyceride 17:1 as internal standard (7). Culture dishes were sonicated in a bath sonicator for 30 s before lipid collection. After centrifugation, the supernatants were retrieved and mixed 300 μL CHCl_3_ and 700 μL of 1% acetic acid to induce phase separation. The organic phase was collected, evaporated in the speed vac (45°C, 20 min), and redissolved in 10 μL CHCl_3_, and the tubes briefly vortexed. To tubes clicked with C171, 40 μl of C171 click mix were added (prepared by mixing 10 μl of 100 mM C171 in 50% methanol with 200 μl 5 mM Cu(I)AcCN_4_BF_4_ in acetonitrile [AcCN] and 800 μl ethanol) while tubes clicked with N_3_Pal, 70 μl of N_3_Pal click mix were added (prepared by mixing 10 μl of 50 mM N_3_Pal in ethanol with 250 μl 5 mM Cu(I)AcCN_4_BF_4_ in AcCN and 750 μl ethanol) followed by sonication for 5 min and incubation at 42°C for 16 h. About 300 μl of CHCl_3_ and 700 μl water were added, and samples were briefly shaken and centrifuged for 5 min at 20,000 *g*. The upper phase was removed, and the lower phase dried in a speed vac as above. About 500 μl of spray buffer (2-propanol/methanol/water 8/5/1 + 10 mM ammonium acetate) was added, and the tubes were sonicated for 5 min and stored at −20°C.

### Determination of lipid recovery

Total lipids from 45,000 unlabeled bEND3 cells (12 identical samples) were isolated as above but using an extraction mix also containing 250 pmol of synthetic pPC 18:0/18:0, pPC 18:1/18:1, pPC 18:2/18:2, pPC 18:3/18:3, pPC 20:4/20:4, and pPC 22:6/22:6. Six samples were processed as usual (sample *a*), and to further six samples (sample *b*), another 250 pmol of all synthetic pPCs was added prior to the click reaction. All samples were click reacted with N_3_Pal before processing continued as usual. Samples were analyzed by MS, and the signal intensities of the homoacyl-pPCs were determined. Recovery as percentage was calculated from the signals according to 100 ∗ *a*/(*b* − *a*).

### Determination of method linearity, detection, and quantification limits

Total lipids from 45,000 unlabeled bEND3 cells were isolated as above and mixed with increasing concentrations of synthetic homoacyl-pPCs and 240 pmol of pPC 31:1. Samples were processed as usual, and employing the N_3_Pal reporter was quantified using the pPC 31:1 internal standard. MS2 signals of the click-reacted lipid (PR2), its diagnostic fragmentation peak upon neutral loss (NL) of 335.26, and of the respective FA were recorded. Five replicate experiments were performed.

### MS analysis

The tubes were sonicated for 5 min, and the dissolved lipids were analyzed. Mass spectra were recorded on a Thermo Q-Exactive Plus spectrometer equipped with a standard heated ESI source using direct injection from a Hamilton syringe driven by a syringe pump under the control of the Tune instrument control software. MS1 spectra (resolution of 280,000) were recorded in 100 *m/z* windows from 250 to 1,200 *m/z* (positive mode) and 950–1,300 *m/z* (negative mode) followed by recording MS/MS spectra (resolution of 280,000) by data-independent acquisition in 1 *m/z* windows from 200 to 1,200 *m/z* (positive mode) and 950–1,300 *m/z* (negative mode).

### MS data analysis

Raw files were converted to mzml files using MSConvert and analyzed using LipidXplorer (8). For identification and quantification of labeled alkyne lipids, molecular fragment query language files were written that identify the species by the presence of a peak corresponding to the expected masses of the labeled lipid class combined with the characteristic NL. Lipids were quantified using the respective internal standard. The applied molecular fragment query language files are provided in the supplemental data.

### Statistical analysis

Statistical differences between sample groups were calculated using GraphPad Prism, version 8.0, software. Two-way ANOVA was followed by Dunnet analysis to correct for multiple comparisons. Family wise significance and confidence level (alpha 0.05; 95% CI) settings were applied. Multiplicity-adjusted *P* values were calculated.

## Results

### The technology

To establish an improved method for choline phospholipid analysis by MS, we reasoned that a chemical modification of the propargylcholine moiety during sample preparation could facilitate the analysis of the lipid. Benefiting from the possibilities of the click reaction (2, 3), we first explored the potential of our recently introduced C171 reporter (6).

**Fig. 1:**
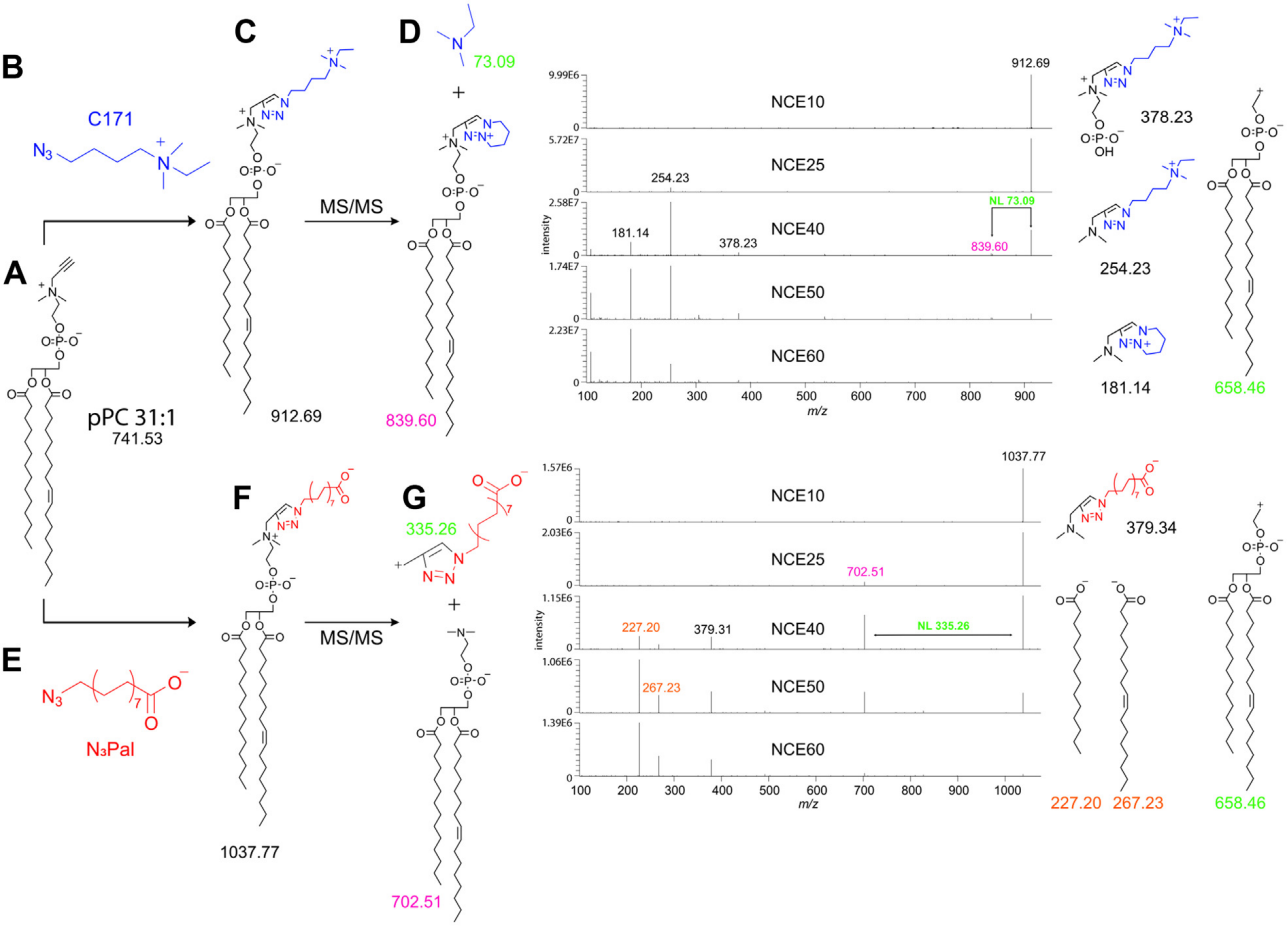
Analysis of pPC by ESI-tandem MS using click-chemistry reporters. Synthetic pPC 31:1 (A) click-reacted with C171 reporter (B) generates a mass-shifted product (C) whose MS2 fragmentation spectra at increasing collision energy in positive mode and the most likely structures (D) are shown. Alternatively, click reaction with N_3_Pal reporter (E) generates a different mass-shifted product (F) whose MS2 fragmentation spectra at increasing normalized collision energy (NCE) in negative mode and the most likely structures (G) are depicted. Magenta numbers indicate the diagnostic fragmentation peaks upon NL and the corresponding molecular structure. Green numbers indicate the molecular structures corresponding to NL fragments. Orange numbers indicate the diagnostic peaks and corresponding FA structures obtained only in negative mode employed by the N_3_Pal reporter method.

The C171 reporter comprises a charged quaternary ammonium group, a linker, and an azido group for reaction with terminal alkynes. For the initial setup of the method, we used a synthetic phosphatidylpropargylcholine, pPC 31:1, featuring myristic acid (FA 14:0) and heptadec-9-enoic acid (FA 17:1) side chains (Fig. 1A). Upon click reaction, the C171 reporter (Fig. 1B) conferred a nominal mass shift of +171 Da to the lipid analyte (Fig. 1C). The positive charge ensured efficient ionization, and at moderate collision energies, the labeled lipid showed the stereotypic NL of 73.09 Da (Fig. 1D) observed before (6). Intriguingly, the introduced positive charge on the bicyclic triazole neighboring that on the quaternary ammonium favors this NL over the commonly observed loss of a positive head group fragment *m/z* 208.07 (1) corresponding to *m/z* 184.07 for regular PC. The NL of 73.09 Da is diagnostic and enables identification of the lipid analyte while providing the sum FA composition. At elevated collision energies, further fragments, specific for the labeled head group moiety, can be detected (Fig. 1D).

To deepen the analysis, an identification of the individual FA side chains would be desirable. To detect fragments of the individual side chains generated in a MS2 setup, we opted for negative-mode MS. Consequently, the use of a different reporter for the click reaction became necessary. Such a reporter should include an azido moiety, ensure adequate ionization of the labeled product to enhance its signal, confer a predictable mass shift to the lipid analyte that allows for its direct identification at the MS1 level, and yield a diagnostic fragmentation pattern at the MS2 level.

N_3_Pal fulfills these requirements (Fig. 1E). Upon click reaction, it confers a nominal mass shift of +296.08 Da to the lipid analyte (Fig. 1F). The negative charge enables efficient ionization in the negative mode, and at moderate collision energies, the labeled lipid showed a stereotypic NL of 335.26 Da (Fig. 1G). Importantly, at moderate and elevated collision energies, side chain-specific fragments (*m/z* 227.20 and 267.23), revealing the identity of the attached FAs (FA 14:0 and FA 17:1, respectively) can be detected along another head group-specific fragment (*m/z* 379.34). This way various lipid classes containing the propargylcholine head group can be analyzed (supplemental Fig. S3). Using our instrumentation and protocol, the method provided a linear range of at least three orders of magnitude, a detection limit of 1 pmol, and a limit of quantification of 4 pmol (supplemental Fig. S4). While delivering an average of 79% analyte recovery for six different pPC species, that of PUFA-containing lipids was found reduced if the FA signal rather than the peak corresponding to the NL was considered (supplemental Fig. S4).

### A proof of concept

To investigate the choline phospholipid metabolism in cells, we chose a labeling strategy employing either the synthetic LpPC 16:0 featuring a palmitic acid side chain, or its ether pendant, LpPC O-16:0 with the corresponding fatty alcohol at the *sn-1* position (supplemental Fig. S1). The brain endothelial cell line bEND3 was incubated with 20 μM of either tracer for 24 h.

**Fig. 2:**
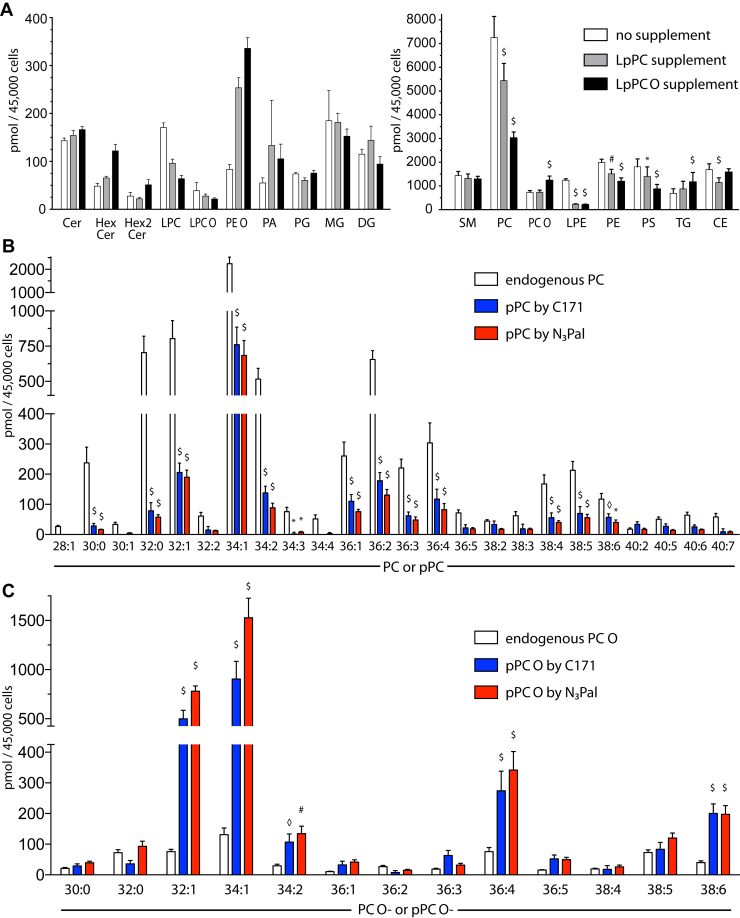
Lipid analysis of labeled cells. (A) Total lipids isolated from bEND3 cells labeled with 20 μM LpPC 16:0, LpPC O-16:0, or carrier for 24 h were quantified by ESI-tandem MS. Major classes of the sphingolipid, glycerophospholipid, and neutral lipid families were quantified using respective internal standards. (B and C) The sum FA composition of the labeled PC (B) or PC O (C) species upon incubation with 20 μM LpPC 16:0 (B) or LpPC O-16:0 (C) for 24 h was analyzed by either the C171 or N_3_Pal reporter method and quantified using the pPC 31:1 internal standard. The unlabeled (endogenous) species were analyzed using the PC 31:1 internal standard. Each species is identified by two numbers: the first is the sum of radyl carbons, and the second is the sum of double bonds present in the two side chains. Lipid amounts are shown as pmol per 45,000 cells and represent means ± 95% CI, N = 7. Lipid species less abundant than 25 pmol under all conditions were omitted from B and C. Two-way ANOVA followed by a Dunnet analysis to correct for multiple comparisons was performed. Adjusted *P* values: ◊*P* < 0.04, ∗*P* < 0.01, #*P* < 0.001, $*P* < 0.0001, all versus control (A, no supplement; B and C, endogenous).

We first determined the effect of labeling on major lipid classes of the sphingolipid, glycerophospholipid, and neutral lipid families (Fig. 2A). Upon incubation with either LpPC 16:0 or LpPC O-16:0, the amounts of unlabeled PC significantly decreased (Fig. 2A and Table 1), indicating a cellular compensation for the surplus of exogenously added propargylcholine phospholipids. Alike the content of PE, LPE, and PS was reduced, while the levels of PC O significantly increased only during incubation with LpPC O-16:0. Addition of LpPC O-16:0 also increased TG, whereas LpPC 16:0 lowered the cholesterol esters (Fig. 2A and supplemental Table S1). The levels of the other tested neutral and glycerophospholipids as well as sphingolipids were not significantly affected.

**Table 1:** Analysis of choline-, ethanolamine-, propargylcholine-containing lipids of labeled cells

	Control (No Supplement)	LpPC 16:0 Supplement	% of Detectable Lipid	LpPC O-16:0 Supplement	% of Detectable Lipid
	pmol			pmol	
LPC	170.7 ± 10.5	96.1 ± 9.4		64.0 ± 7.3	
LPC O	39.1 ± 18.2	27.6 ± 4.6		21.4 ± 2.7	
LPE	1,250.0 ± 78.2	228.3 ± 28.5		202.1 ± 24.5	
PC	7,257.4 ± 959.0	5,439.7 ± 781.8		3,026.5 ± 268.5	
PC O	731.8 ± 82.2	734.8 ± 96.7		1,240.1 ± 190.6	
PE	1,991.1 ± 147.3	1,511.3 ± 204.9		1,192.6 ± 159.7	
PE O	83.5 ± 10.9	253.7 ± 22.9		335.9 ± 24.1	
SM	1,437.1 ± 185.9	1,324.4 ± 194.9		1,290.1 ± 127.7	
pPC	0 ± 0.0	1,728.1 ± 285.9	85.6	519.7 ± 79.2	12.3
pPC O	0 ± 0.0	15.8 ± 14.3	0.8	3,511.7 ± 511.2	83.0
pSM	0 ± 0.0	274.8 ± 45.6	13.6	200.4 ± 26.8	4.7

LpPC, lyso-propargyl-PC; LPC, lysophosphatidylcholine; LPC O, ether lysophosphatidylcholine; LPE, lysophosphatidylethanolamine; PC, phosphatidylcholine; PC O, ether phosphatidylcholine; PE, phosphatidylethanolamine; PE O, ether phosphatidylethanolamine; pPC, propargyl phosphatidylcholine; pPC O, ether propargyl phosphatidylcholine; pSM, propargyl sphingomyelin; SM, sphingomyelin.

Total lipids isolated from bEND3 cells labeled with 20 μM LpPC 16:0, LpPC O-16:0, or carrier for 24 h were quantified by ESI-tandem MS. Choline-, ethanolamine-, and propargylcholine-containing lipids were quantified using the internal standards PC 31:1, SM 17:0, PE 31:1, and pPC 31:1, respectively. Propargylcholine lipids were analyzed by the azidopalmitate reporter method. Lipid amounts are shown as picomol per 45,000 cells and represent means ± SD, N = 7. The data of the unlabeled lipids correspond to the graphs depicted in Fig. 2A. The specificity of labeling (preference for the primary target lipid class) has been calculated and is expressed as percentage of the total detectable label.

Next, we aimed to elucidate the metabolic fate of the labeling lipids. Upon uptake, LpPC 16:0 or LpPC O-16:0 underwent cellular acylation to yield pPC or pPC O, respectively (Table 1). An analysis of the pPC species generated from the LpPC 16:0 tracer was performed by applying the C171 or the N_3_Pal reporter method (Fig. 2B). Comparing the species distribution of the unlabeled endogenous PCs with that of the labeled pPC pool, a clear correlation emerged. The most prevalent species showed a range of 32–36 carbons in their side chains, and 34 carbons were most abundant. The degree of FA saturation also matched well between both pools. Detection of pPC species by the C171 versus N_3_Pal reporter methods often yielded comparable amounts (Fig. 2B and Table 2, species columns).

**Table 2:** Species and subspecies distribution of the propargylcholine-labeled PC pool

Species			Subspecies				
	pmol by C171 (NL 73.09)	pmol by N_3_Pal (NL 335.26)	FA1	FA2	pmol by N_3_Pal (Sum of Subspecies; FA Peaks)	pmol by N_3_Pal (Individual Subspecies; FA Peaks)	% of All Subspecies
pPC 30:0	**28.2 ± 9.2**	*16.4 ± 2.0*	FA 16:0	FA 14:0	*19.3 ± 2.8*	*19.3 ± 2.8*	*100 ± 0.0*
pPC 30:1	**0.0 ± 0.0**	*4.9 ± 1.9*	FA 16:1	FA 14:0	*3.1 ± 0.8*	*3.1 ± 0.8*	*100 ± 0.0*
pPC 32:0	**79.0 ± 29.0**	*60.8 ± 8.6*	FA 18:0	FA 14:0	*82.3 ± 5.3*	*2.3 ± 0.7*	*2.8 ± 0.9*
			FA 16:0	FA 16:0		*80.0 ± 5.6*	*97.2 ± 0.9*
pPC 32:1	**205.9 ± 33.2**	*188.2 ± 34.2*	FA 18:1	FA 14:0	*194.0 ± 16.8*	*10.3 ± 2.1*	*5.3 ± 0.8*
			FA 16:0	FA 16:1		*183.6 ± 15.5*	*94.7 ± 0.8*
pPC 32:2	**14.7 ± 12.7**	*12.7 ± 2.6*	FA 18:2	FA 14:0	*12.5 ± 2.4*	*0.8 ± 0.3*	*6.7 ± 2.6*
			FA 16:0	FA 16:2		*3.8 ± 0.3*	*31.4 ± 6.4*
			FA 16:1	FA 16:1		*7.8 ± 2.3*	*61.9 ± 7.7*
pPC 34:1	**760.3 ± 134.6**	*677.6 ± 129.4*	FA 20:1	FA 14:0	*706.5 ± 91.3*	*0.4 ± 0.5*	*0.1 ± 0.1*
			FA 18:0	FA 16:1		*20.0 ± 4.2*	*2.9 ± 0.5*
			FA 18:1	FA 16:0		*686.1 ± 89.5*	*97.1 ± 0.5*
pPC 34:2	**137.9 ± 24.4**	*89.5 ± 20.9*	FA 18:1	FA 16:1	*96.7 ± 9.0*	*55.6 ± 8.9*	*57.3 ± 4.9*
			FA 18:2	FA 16:0		*41.0 ± 3.7*	*42.7 ± 4.9*
pPC 34:3	**2.3 ± 6.1**	*7.6 ± 3.3*	FA 20:3	FA 14:0	*20.5 ± 4.2*	*10.9 ± 3.9*	*51.8 ± 8.9*
			FA 18:1	FA 16:2		*1.7 ± 0.5*	*8.8 ± 2.9*
			FA 18:2	FA 16:1		*3.7 ± 0.5*	*18.9 ± 4.5*
			FA 18:3	FA 16:0		*4.1 ± 0.5*	*20.5 ± 3.7*
pPC 34:4	**0.0 ± 0.0**	*2.8 ± 5.1*	FA 20:4	FA 14:0	*25.6 ± 7.8*	*25.6 ± 7.8*	*100.0 ± 0.0*
pPC 36:1	**110.2 ± 24.0**	*73.5 ± 9.6*	FA 20:1	FA 16:0	*91.8 ± 9.0*	*14.2 ± 2.2*	*15.5 ± 2.6*
			FA 18:0	FA 18:1		*77.7 ± 8.9*	*84.5 ± 2.6*
pPC 36:2	**178.2 ± 29.2**	*125.1 ± 21.8*	FA 20:1	FA 16:1	*133.6 ± 26.9*	*4.3 ± 0.3*	*3.3 ± 0.5*
			FA 20:2	FA 16:0		*7.1 ± 1.5*	*5.5 ± 1.3*
			FA 18:0	FA 18:2		*12.9 ± 2.8*	*9.7 ± 0.7*
			FA 18:1	FA 18:1		*109.3 ± 23.6*	*81.6 ± 2.0*
pPC 36:3	**62.5 ± 13.1**	*48.4 ± 14.2*	FA 20:2	FA 16:1	*48.5 ± 3.5*	*1.7 ± 0.2*	*3.5 ± 0.5*
			FA 20:3	FA 16:0		*27.0 ± 3.6*	*55.6 ± 4.3*
			FA 18:0	FA 18:3		*1.5 ± 0.5*	*3.1 ± 1.2*
			FA 18:1	FA 18:2		*18.3 ± 1.4*	*37.8 ± 3.5*
pPC 36:4	**117.1 ± 35.5**	*85.4 ± 29.5*	FA 22:4	FA 14:0	*71.7 ± 10.6*	*3.9 ± 4.7*	*5.2 ± 5.7*
			FA 20:3	FA 16:1		*2.8 ± 0.4*	*4.1 ± 1.2*
			FA 20:4	FA 16:0		*60.9 ± 10.1*	*84.9 ± 5.4*
			FA 18:1	FA 18:3		*3.4 ± 0.5*	*4.8 ± 1.1*
			FA 18:2	FA 18:2		*0.7 ± 0.2*	*1.1 ± 0.3*
pPC 36:5	**22.0 ± 11.2**	*19.2 ± 6.3*	FA 22:5	FA 14:0	*12.5 ± 2.2*	*0.9 ± 0.4*	*7.5 ± 3.1*
			FA 20:4	FA 16:1		*3.1 ± 0.5*	*24.8 ± 2.8*
			FA 20:5	FA 16:0		*8.5 ± 1.7*	*67.7 ± 4.5*
pPC 38:2	**32.8 ± 13.1**	*17.5 ± 2.7*	FA 22:0	FA 16:1	*25.2 ± 2.3*	*6.6 ± 0.9*	*26.2 ± 3.0*
			FA 22:2	FA 16:0		*3.4 ± 0.3*	*13.4 ± 1.7*
			FA 20:1	FA 18:1		*12.6 ± 1.5*	*50.0 ± 2.4*
			FA 20:2	FA 18:0		*2.6 ± 0.5*	*10.3 ± 1.5*
pPC 38:3	**19.3 ± 15.8**	*17.1 ± 3.9*	FA 22:3	FA 16:0	*20.7 ± 1.5*	*3.8 ± 0.9*	*18.3 ± 3.9*
			FA 20:1	FA 18:2		*1.4 ± 0.5*	*6.9 ± 2.3*
			FA 20:2	FA 18:1		*5.1 ± 0.7*	*24.9 ± 3.8*
			FA 20:3	FA 18:0		*10.3 ± 1.0*	*49.9 ± 3.0*
pPC 38:4	**56.0 ± 17.1**	*40.6 ± 8.8*	FA 22:4	FA 16:0	*38.1 ± 5.0*	*9.8 ± 2.1*	*25.5 ± 3.4*
			FA 20:2	FA 18:2		*0.2 ± 0.2*	*0.6 ± 0.7*
			FA 20:3	FA 18:1		*10.8 ± 1.5*	*28.5 ± 3.3*
			FA 20:4	FA 18:0		*17.4 ± 2.5*	*45.5 ± 2.0*
pPC 38:5	**70.1 ± 24.5**	*57.3 ± 15.1*	FA 22:4	FA 16:1	*48.1 ± 7.6*	*2.1 ± 0.8*	*4.4 ± 1.4*
			FA 22:5	FA 16:0		*23.8 ± 5.0*	*49.1 ± 4.0*
			FA 20:3	FA 18:2		*0.5 ± 0.4*	*1.0 ± 0.8*
			FA 20:4	FA 18:1		*18.1 ± 2.3*	*39.0 ± 3.6*
			FA 20:5	FA 18:0		*3.6 ± 1.0*	*7.4 ± 1.4*
pPC 38:6	**56.9 ± 13.1**	*42.5 ± 11.2*	FA 22:5	FA 16:1	*28.6 ± 5.0*	*3.0 ± 1.2*	*10.2 ± 2.4*
			FA 22:6	FA 16:0		*19.9 ± 3.7*	*69.8 ± 3.7*
			FA 20:4	FA 18:2		*2.1 ± 0.3*	*7.5 ± 1.8*
			FA 20:5	FA 18:1		*3.6 ± 0.7*	*12.6 ± 1.4*
pPC 40:2	**33.8 ± 9.2**	*16.9 ± 4.7*	FA 24:1	FA 16:1	*29.2 ± 5.1*	*4.8 ± 0.8*	*16.4 ± 1.8*
			FA 22:0	FA 18:2		*1.4 ± 0.4*	*4.9 ± 1.4*
			FA 22:1	FA 18:1		*23.0 ± 4.3*	*78.8 ± 2.9*
pPC 40:5	**27.2 ± 8.7**	*13.7 ± 4.6*	FA 22:3	FA 18:2	*15.4 ± 2.5*	*0.2 ± 0.2*	*1.3 ± 1.7*
			FA 22:4	FA 18:1		*5.9 ± 2.3*	*37.6 ± 7.3*
			FA 22:5	FA 18:0		*7.3 ± 0.8*	*47.7 ± 4.4*
			FA 20:1	FA 20:4		*2.0 ± 0.5*	*13.4 ± 4.1*
pPC 40:6	**25.6 ± 7.2**	*15.8 ± 3.4*	FA 22:5	FA 18:1	*17.2 ± 3.5*	*11.6 ± 3.4*	*66.6 ± 5.3*
			FA 22:6	FA 18:0		*5.6 ± 0.6*	*33.4 ± 5.3*
pPC 40:7	**8.8 ± 11.1**	*7.4 ± 4.2*	FA 22:5	FA 18:2	*9.0 ± 1.4*	*0.9 ± 0.4*	*9.8 ± 2.8*
			FA 22:6	FA 18:1		*7.4 ± 1.0*	*82.5 ± 6.5*
			FA 20:3	FA 20:4		*0.7 ± 0.4*	*7.8 ± 4.1*
total	**2,048 ± 317**	*1,641 ± 279*			*1,750 ± 151*		

PC, phosphatidylcholine; NL, neutral loss; N_3_Pal, azidopalmitate; pPC, propargyl phosphatidylcholine.

Total lipids isolated from bEND3 cells labeled with 20 μM lyso-propargyl-PC 16:0 for 24 h were analyzed using either the C171 (Bold values) or the N_3_Pal (Italic values) reporter method. Each lipid species was detected, identified as sum FA composition and quantified using either the NL73.09 or the NL335.26 peak, and pPC 31:1 as internal standard by either method (species columns; corresponding to data in Fig. 2B). Only the N_3_Pal reporter method delivered the subspecies composition (subspecies columns) revealing the identity of the two FAs. The sum of the peaks corresponding to both FA fragments was used to quantify the subspecies and the subspecies' proportion on all subspecies. Lipid amounts are shown as pmol per 45,000 cells and represent means ± SD; N = 7. Lipid species less abundant than 25 pmol under all conditions depicted in Fig. 2B were omitted.

The main advantage of the N_3_Pal-based detection over the C171 reporter method, however, is that it delivers subspecies information by identifying the two FA side chains, in addition to the sum FA composition (Table 2, subspecies columns). Out of the reported 23 pPC species, only the N_3_Pal-based detection revealed that 20 species contained subspecies, whereas 3 species did not show subspecies. Up to five subspecies could be detected for two species (pPC 36:4 and pPC 38:5). When analyzing the abundance of each FA among all 70 detected pPC subspecies, the generally most frequent palmitate, oleate, palmitoylate, and stearate ranked highest, and together comprised 83.4% of all FAs (Table 3). Arachidonate ranked fifth (3.7% of all FAs) and was found in eight subspecies. Together, all PUFAs encompassed 12.4% of all FAs and showed the widest distribution in the pool. Given the fact that the employed LpPC 16:0 tracer featured a palmitate side chain and that out of the 70 detected pPC subspecies, 53 (corresponding to 554 pmol/45,000 cells or 32% of all pPC molecules) did not contain a palmitate, a substantial lipid remodeling within the pool became evident.

**Table 3:** FA distribution in the propargylcholine-labeled PC pool

	Abundance Ranking	pmol in 70 Subspecies	% of Total	Found in x of 140 Positions
FA 16:0	1	1,276.3	36.5	18
FA 18:1	2	1,169.8	33.4	18
FA 16:1	3	310	8.9	15
FA 18:0	4	161.2	4.6	11
FA 20:4	5	129.9	3.7	8
FA 18:2	6	84.8	2.4	14
FA 14:0	7	77.5	2.2	10
FA 20:3	8	63	1.8	7
FA 22:5	9	47.5	1.4	6
FA 20:1	10	34.9	1.0	6
FA 22:6	11	32.9	0.9	3
FA 22:1	12	23	0.7	1
FA 22:4	13	21.7	0.6	4
FA 20:2	14	16.7	0.5	5
FA 20:5	15	15.7	0.4	3
FA 18:3	16	9	0.3	3
FA 22:0	17	8	0.2	2
FA 16:2	18	5.5	0.2	2
FA 24:1	19	4.8	0.1	1
FA 22:3	20	4	0.1	2
FA 22:2	21	3.4	0.1	1
SFA		1,523	43.5	41
MUFA		1,543	44.1	41
PUFA		434	12.4	58

PC, phosphatidylcholine; SFA, saturated fatty acid.

Total lipids isolated from bEND3 cells labeled with 20 μM lyso-propargyl-PC 16:0 for 24 h were analyzed using the azidopalmitate reporter method. Each lipid species was identified using the NL335.26 peak and the side chains by the respective FA fragment peaks. The two FAs were quantified as half of their sum, and the FA fragment peaks generated from pPC 31:1 served as internal standard. A total of 70 pPC subspecies carrying FA side chains at 140 possible positions were analyzed. FA amounts are shown as pmol per 45,000 cells and represent means; N = 7. The data correspond to the subspecies columns in Table 2.

If analyzing a sample using the N_3_Pal reporter method, the quantification of the peak corresponding to the fragment after an NL of 335.26 Da provides a sensitive means of detection (Fig. 1G). As this peak is specific and abundant, it represents the favorable way to quantify pPC species using an internal pPC standard. Alternatively, the signal intensities of the peaks corresponding to the fragmented FA side chains may be used. Comparing the two quantification ways, a generally good agreement between both approaches was found (Table 2). Hence, the total pPC content in 45,000 cells was determined as 1,641 or 1,750 pmol for the method based on NL 335.26 or the FA peaks, respectively. Both numbers were in good agreement with 2048 pmol, the value obtained from the C171 reporter method (Table 2, bottom).

Next, we investigated the metabolic fate of the LpPC O-16:0 tracer. Also here the distribution of the labeled pPC O species generated by cellular metabolism showed a clear correlation to that of the unlabeled endogenous PC O (Fig. 2C and supplemental Table S2). Again, the side-chain carbon range and saturation degree matched well between both pools. The PC O pool contained PUFAs at a frequency of 28%. At least for the five most abundant pPC O species, a neglectable occurrence of fatty alcohol vinylation was found, rendering the identified metabolites labeled plasmanyl species. Detection of the labeled pPC O species by the N_3_Pal reporter method generally showed a higher sensitivity than analysis by the C171-based method. Remarkably, either method reported significantly more pPC O than PC O, demonstrating a larger pool size of the labeled versus the unlabeled ether PCs upon tracer supplementation (supplemental Table S2, bottom). As this deviated from the data obtained for the pPCs, it pointed to differences in the metabolism of ether versus nonether PCs (Table 1).

Comparing data on the LpPC 16:0 and LpPC O-16:0 tracers, we found that both precursors directed the propargylcholine label to their primary target lipid class with similarly high efficiencies (Table 1, bottom). At the investigated time point, about 85% of all detectable labels were found in the respective target lipid class, whereas about 15% became transferred to other lipids including SM. Besides labeled propargyl sphingomyelin (pSM), we also found the labeled ether/nonether counterpart of the primary target class, all indicative of lipid remodeling that likely involved head group exchange. However, a different remodeling probability for metabolites generated from LpPC 16:0 or LpPC O-16:0 became apparent. While LpPC 16:0-derived pPC showed a relatively high propensity for propargylcholine transfer to SM yielding pSM (13.6%) and little conversion into pPC O (0.8%), the LpPC O-16:0-derived pPC O was more likely to donate its head group for pPC formation (12.3%) and less so for pSM synthesis (4.7%). Taking in addition the different pool sizes of the primarily labeled metabolites (1,728 vs. 3,512 pmol/45,000 cells for pPC or pPC O, respectively) and the endogenous pools (7,257 vs. 732 pmol/45,000 cells for PC or PC O, respectively) into consideration (Table 1), the differences in ether and nonether PC metabolism were further emphasized. According to our data, a single bEND3 cell contained 161 fmol PC and 16 fmol PC O. Labeling by LpPC 16:0 or LpPC O-16:0 yielded 38 fmol pPC or 78 fmol pPC O per cell.

## Discussion

Propargylcholine labeling of choline-containing lipids has been proven a valuable tool to investigate the cell biology and metabolism of PC (1). The versatility of the alkyne tag in combination with advanced click reporters, detection technologies, and instrumentation has opened new possibilities in lipid research (9). However, the particular developments in alkyne lipid tracing by MS have thus far focused on side chain-tagged alkyne lipids (6). For an alkyne head group such as propargylcholine, the potential of the new methodologies had not been explored.

Propargylcholine-containing lipids have been analyzed by MS using scans for [M + H]^+^ ions in positive ion mode that are a precursor of *m/z* 208.1 (1). This approach has some intrinsic limitations. When unfractionated lipid extracts are continuously infused into the ESI source on a quadrupole, an overlap of various parent ions occurs. During MS2 scanning to detect the *m/z* 208.1 fragment, such overlap can cause problems, as this fragment is head group specific but carries no intrinsic information on the side chains.

Our strategy employing click reaction and the C171 reporter overcomes this shortcoming. In MS2 analysis, this reporter gives a characteristic NL so that the backbone of the labeled lipid still appears as a charged fragment. This improves the specificity of the analysis because the analyzed lipid is defined by two specific ions enabling resolution of isobaric species in MS2, which would not be possible with a charged reporter ion such as the 208.1 Da propargylcholine head group (6). In addition, the quantification benefits from the high sensitivity of MS2 scanning in 1 Da windows. With unfractionated lipid extracts, minor species occasionally fail to give peaks in MS1, whereas in MS2, both the precursor and the fragments are detected, allowing for unequivocal identification and quantification. However, both the conventional strategy based on the 208.1 Da reporter ion and the C171 reporter approach employing the NL of 73.1 reliably deliver only the sum FA composition of the lipid analyte.

Our novel method employing click reaction and the N_3_Pal reporter also overcomes this limitation. While maintaining the ionization ease in the negative ion mode applied here, it benefits from all advantages achieved with the C171 reporter and more. The nominal mass shift of +296 Da by the N_3_Pal reporter relocates the ion of the analyte to a portion of the MS1 spectrum that is hardly occupied and thus effectively reduces parent ion overlap. During MS2 analysis, the N_3_Pal method also provides a characteristic NL preserving the lipid backbone information in the detected fragment. The signal for the NL 335 seen here is about 3-fold stronger than that obtained for the NL 73 detected for the C171 reporter method using positive mode. Both methods profit from the high sensitivity of MS2 scanning in 1 Da windows. In a negative mode MS2 analysis, the N_3_Pal method also shows advantages over a possible direct detection of unclicked pPCs as acetate counterion adducts with methyl group elimination (10). While ionization efficiencies are comparable, the N_3_Pal method reliably delivers far more intense parent peaks in MS2.

Importantly, the lipid analyzed by the N_3_Pal method is defined by two specific ions with high diagnostic power and in addition by FA-specific fragments, revealing the identity of the side chains. However, no information on *sn-1/sn-2* placement or positioning of double bonds within the side chain is obtained. Yet, as cellular metabolism is unlikely to change the *sn-1* linkage of the fatty alcohol in ether lipids, the liberated FA can be assumed to originate from the *sn-2* position in the case of ether lipids.

For labeling of the choline-containing lipid pool methods using D9-choline and stable isotope tagging have proven invaluable (11, 12, 13). An alternative strategy employs propargylcholine (1). Our approach relates to the latter but also employs click reaction and dedicated reporters. In addition, we chose to use LpPCs as labeled precursors as these tracers represent a good compromise between precursor solubility and labeling specificity. Using an intermediate concentration of LpPC 16:0 or LpPC O-16:0, a superior labeling of the respective target lipid class pPC or pPC O with high specificity and efficiency was achieved.

As the cells take up and metabolize these precursors, they exert parallel adjustments to their lipidome. Unsurprisingly, the pool of endogenous PC is affected strongest and reduced accordingly (14). The precision of the underlying regulatory mechanisms is intriguing, and we find a superb compensation, illustrated by excellent numerical matching of pool size adaptations. For one parameter, the cells appear to adjust their membrane composition for steady proportions of the different lipid head groups. As observed before, propargylcholine is well accepted by cellular metabolism and substitutes effectively for the choline moiety in lipids (1). Accordingly, the levels of endogenous PC are reduced by both the labeled pPC and its ether pendant pPC O to accommodate the surplus of propargylcholine head groups. Here, the nature of the *sn-1* side chain (FA or fatty alcohol) appears to play a secondary role. However, certain flexibility for gross adjustments in head group composition may exist as we also find reductions in ethanolamine-containing lipids (endogenous LPE or PE) and PS for either precursor treatment. All other tested lipid classes displayed no changes with the notable exception of TG and cholesteryl ester, the relevance of the latter remains unclear.

Apart from the head group composition, cells are also known to precisely fine tune the FA profile within their lipidome. When comparing the labeled metabolites of LpPC 16:0 or LpPC O-16:0 to the endogenous lipid pool, we find a well-matching pattern of side-chain length and saturation. Not only similar species were identified but also the ranking of their abundance was similar. Although both tracers contain a saturated tail of 16 carbons, a great variety of side chains were found in the metabolites. For labeled pPC, a third of all species was not containing palmitate, and hence surely derived from lipid remodeling. While this again demonstrates the great acceptance of the propargyl label by the involved enzymatic machinery, it also shows the extent of lipid remodeling. Palmitate is generally considered the most abundant saturated FA and is usually found at the *sn-1* position in membrane lipids. Hence, acylation of the LpPC 16:0 tracer would right away yield a very common lipid species, and yet, the cells spend considerable efforts to further modify at least 32% of these initial metabolites during remodeling. That way the cells maintain a specific side-chain composition within each lipid class that is paralleled by a certain head group distribution within the whole lipidome. Noteworthy, the profile of labeled pPC species observed in our experiments does not indicate whether it originates from head group or side-chain remodeling. Likely, both activities exerted by phospholipases C/D or A1/A2, respectively, will occur within our extended experimental time frame.

Comparing the remodeling of LpPC 16:0-derived and LpPC O-16:0-derived lipids, some differences in ether versus nonether lipids became apparent. When labeling cells under equal conditions, both precursors labeled their primary target lipid class with a superb efficiency of ∼85%. However, the total amount of metabolites from LpPC 16:0 was twofold lower than that from LpPC O-16:0. Conversely, the pool size of endogenous PC in bEND3 cells is tenfold higher than that of its ether pendant PC O. This led to very different proportions of labeled versus endogenous lipids in both classes. While a quarter of all PC molecules became labeled, one endogenous PC O was matched by three labeled PC O molecules. If assuming a similar rate of precursor uptake and primary acylation, a longer dwell time of the propargyl label within the ether versus the nonether PC pool is indicated. This is in accordance with the calculated long half-lives of ether lipids in neuronal cells and whole brain (15, 16) but does not exclude the existence of short-lived subpopulations (17).

When analyzing the label transfer away from the primary metabolite pool to other lipid classes, a comparable overall frequency (15% for pPC vs. 17% for pPC O) of head group exchange was found. However, some differences were observed. Head group transfer yielding labeled pSM was threefold more frequent for labeled pPC than pPC O, despite the latter being twofold more abundant. This may indicate that the involved enzymes, such as the SM synthases (18, 19), prefer pPC over pPC O as head group donor. This notion is in line with the idea of a higher stability of the propargyl label within the ether versus the nonether PC pool. Finally, we noted a profoundly higher occurrence of PUFAs in the pPC O (28%) versus the pPC (12%) pool of the analyzed bEND3 cells. This may relate to the described function of ether lipids to act as reservoirs for PUFAs (20). Because PUFAs tend to release carbon dioxide during fragmentation and hence escape detection, our analysis likely underrepresents their general abundance within both pools (21, 22, 23, 24). Indeed, when evaluating several synthetic pPC species, our instrumentation showed a profoundly reduced response to those lipids that contained two PUFAs, and this effect increased with the number of double bonds.

Taken together, the biological data presented here open a quantitative view on the choline phospholipid metabolism in bEND3 cells. They reveal differences in metabolism of PC and ether PC while demonstrating the power of the newly introduced tracing tools. The novel method developed here will greatly facilitate further studies in the field.

### Data availability

Data are available from the authors on reasonable request.

## Conflict of interest

The authors declare that they have no conflicts of interest with the contents of this article.
