# Supplementary material for: An advanced method for propargylcholine phospholipid detection by direct-infusion MS
Source: J Lipid Res. 2021 Jan 13;62:100022. doi: 10.1016/j.jlr.2021.100022 (PMC7900581; doi:10.1016/j.jlr.2021.100022)
Supplement: Supplementary material [file mmc1.pdf]

Supplemental material

to

An advanced method for propargylcholine phospholipid detection by direct-infusion mass spectrometry

by

Mohamed H. Yaghmour, Christoph Thiele, and Lars Kuerschner

including:

Supplemental methods

Supplemental Figure S1

Supplemental Figure S2

Supplemental Figure S3

Supplemental Figure S4

Supplemental Table S1

Supplemental Table S2

Supplemental methods

The following mfql files have been used for data analysis.

```
QUERYNAME = pPCNL73; #search for propargylcholine-containing PCs upon click-reaction to C171
DEFINE PR1 = 'C[32..64] H[50..130] N[5] P[1] O[8]' WITH DBR = (3.5,15), CHG = 1; #first precursor ion (click-reacted to C171)
DEFINE PR2 = 'C[32..64] H[50..130] N[5] P[1] O[8]' WITH DBR = (3.5,15), CHG = 1; #second precursor ion (click-reacted to C171)
DEFINE NL1 = 'C4 H11 N1' WITH CHG = 0; #the characteristic neutral loss
DEFINE PR3 ='C9 H17 N4' WITH CHG = 1; #head group specific fragment 1
DEFINE PR4 ='C13 H28 N5' WITH CHG = 1; #head group specific fragment 2

IDENTIFY
PR1 IN MS1+
AND PR2 IN MS2+
AND NL1 IN MS2+
AND PR3 IN MS2+
AND PR4 IN MS2+

SUCHTHAT
isEven(PR1.chemsc[C])
AND isOdd(PR1.chemsc[H])
AND PR1.chemsc == PR2.chemsc

REPORT
NAME = "pPC [%d:%d]" % ((PR1.chemsc[C] - 18), (PR1.chemsc[db] - 4)); #the name of the unclicked lipid
chemsc = PR1.chemsc; #its chemical sum formula
C = "%d" %  (PR1.chemsc[C] - 18); #the number of carbons in the side chains excluding those of C171
db = "%d" %  (PR1.chemsc[db] - 2); #the number of double bonds in the side chains excluding those of C171
MASS = "%4.4f" % "(PR1.mass)"; #the mass of PR1 with four digits
ERROR = "%2.2f ppm" % "(PR1.errppm)"; #the mass error in ppm
PR2 = PR2.intensity; #intensity of the parent in MS2
INTNL1 = NL1.intensity; ; #intensity of the characteristic NL73 product ion in MS2. Used for all subsequent calculations
```

```
QUERYNAME = pPCONL73; #search for propargylcholine-containing ether-linked PCs upon click-reaction to C171
DEFINE PR1 = 'C[32..64] H[50..130] N[5] P[1] O[7]' WITH DBR = (3.5,15), CHG = 1; #first precursor ion (click-reacted to C171)
DEFINE PR2 = 'C[32..64] H[50..130] N[5] P[1] O[7]' WITH DBR = (3.5,15), CHG = 1; #second precursor ion (click-reacted to C171)
DEFINE NL1 = 'C4 H11 N1' WITH CHG = 0; #the characteristic neutral loss
DEFINE PR3 ='C9 H17 N4' WITH CHG = 1; #head group specific fragment 1
DEFINE PR4 ='C13 H28 N5' WITH CHG = 1; #head group specific fragment 2

IDENTIFY
PR1 IN MS1+
AND PR2 IN MS2+
```

```
AND NL1 IN MS2+
AND PR3 IN MS2+
AND PR4 IN MS2+

SUCHTHAT
isEven(PR1.chemsc[C])
AND isOdd(PR1.chemsc[H])
AND PR1.chemsc == PR2.chemsc

REPORT
NAME = "epPC [%d:%d]" % ((PR1.chemsc[C] - 18), (PR1.chemsc[db] - 3));
#the name of the unclicked lipid
chemsc = PR1.chemsc; #its chemical sum formula
C = "%d" %  (PR1.chemsc[C] - 18); #the number of carbons in the side
chains excluding those of C171
db = "%d" %  (PR1.chemsc[db] - 2); #the number of double bonds in the
side chains excluding those of C171
MASS = "%4.4f" % "(PR1.mass)"; #the mass of PR1 with four digits
ERROR = "%2.2f ppm" % "(PR1.errppm)"; #the mass error in ppm
PR2 = PR2.intensity; #intensity of the parent in MS2
INTNL1 = NL1.intensity; ; #intensity of the characteristic NL73
product ion in MS2. Used for all subsequent calculations
```

QUERYNAME = **pPCNL335**; #search for *propargylcholine-containing PCs upon click-reaction* ***to N3Pal***

```
DEFINE PR1 = 'C[54..72] H[80..150]  P[1] N4 O[10]' WITH DBR =
(3.5,25), CHG = -1; #first precursor ion (click-reacted to N3Pal)
DEFINE PR2 =  'C[54..72] H[80..150]  P[1] N4 O[10]' WITH DBR =
(3.5,25), CHG = -1; #second precursor ion (click-reacted to N3Pal)
DEFINE NL1 = 'C19 H33 N3 O[2]' WITH CHG = 0; #the characteristic
neutral loss
DEFINE FA1 = 'C[12..24] H[19..47] O[2]' WITH DBR = (0.5,8), CHG = -1;
#the first fatty acid
DEFINE FA2 =  'C[12..24] H[19..47] O[2]' WITH DBR = (0.5,8), CHG = -
1; #the second fatty acid

IDENTIFY
PR1 IN MS1-
AND PR2 IN MS2-
AND NL1 IN MS2-
AND FA1 IN MS2-
AND FA2 IN MS2-

SUCHTHAT
isEven(PR1.chemsc[C]) AND
isEven(FA1.chemsc[C]) AND
PR1.chemsc == PR2.chemsc AND
FA1.chemsc + FA2.chemsc + NL1.chemsc +  'C7 H15 N1 P1 O4' ==
PR1.chemsc

REPORT
NAME = "pPC(%d:%d)" % ((PR1.chemsc[C] - 26), (PR1.chemsc[db] - 6));
#the name of the unclicked lipid
FA1 =  "FA(%d:%d)" % ((FA1.chemsc[C]), (FA1.chemsc[db] - 1)); #the
name of the first fatty acid
FA2 =  "FA(%d:%d)" % ((FA2.chemsc[C]), (FA2.chemsc[db] - 1)); #the
name of the second fatty acid
chemsc = PR1.chemsc; #the chemical sum formula of the lipid
C = "%d" %  (PR1.chemsc[C] - 26); #the number of carbons in the side
chains excluding those of N3Pal
```

```
db = "%d" %  (PR1.chemsc[db] - 6); #the number of double bonds in the side chains excluding those of N3Pal
MASS = "%4.4f" % "(PR1.mass)"; #the mass of PR1 with four digits
ERROR = "%2.2f ppm" % "(PR1.errppm)"; #the mass error in ppm
INTNL1 = NL1.intensity; #intensity of the characteristic NL335 product ion in MS2
PR2 = PR2.intensity; #intensity of the parent in MS2
allFAs = sumIntensity(FA1.intensity, FA2.intensity); ; #intensity of the summarized side chains in MS2
```

```
QUERYNAME = plasmAnylpPCNL335; #search for propargylcholine-containing ether-linked PCs upon click-reaction to N3Pal
DEFINE PR1 = 'C[54..66] H[101..131]  P[1] N4 O[9]' WITH DBR = (3.5,25), CHG = -1; #first precursor ion (click-reacted to N3Pal)
DEFINE PR2 = 'C[54..66] H[101..131]  P[1] N4 O[9]' WITH DBR = (3.5,25), CHG = -1; #second precursor ion (click-reacted to N3Pal)
DEFINE NL1 = 'C19 H33 N3 O[2]' WITH CHG = 0; #the characteristic neutral loss
DEFINE FA1 = 'C[12..24] H[19..47] O[2]' WITH DBR = (0.5,8), CHG = -1; #the only fatty acid

IDENTIFY
PR1 IN MS1-
AND PR2 IN MS2-
AND NL1 IN MS2-
AND FA1 IN MS2-

SUCHTHAT
isEven(PR1.chemsc[C]) AND
PR1.chemsc == PR2.chemsc AND
FA1.chemsc + NL1.chemsc +  'C23 H48 N1 P1 O5' == PR1.chemsc

REPORT
NAME = "etherAnyl-pPC(%d:%d)" % ((PR1.chemsc[C] - 26), (PR1.chemsc[db] - 5)); #the name of the unclicked lipid
FA1 =  "FA(%d:%d)" % ((FA1.chemsc[C]), (FA1.chemsc[db] - 1)); #the name of the only fatty acid
chemsc = PR1.chemsc; #the chemical sum formula of the lipid
C = "%d" %  (PR1.chemsc[C] - 26); #the number of carbons in the side chains excluding those of N3Pal
db = "%d" %  (PR1.chemsc[db] - 4); #the number of double bonds in the side chains excluding those of N3Pal
MASS = "%4.4f" % "(PR1.mass)"; #the mass of PR1 with four digits
ERROR = "%2.2f ppm" % "(PR1.errppm)"; #the mass error in ppm
INTNL1 = NL1.intensity; #intensity of the characteristic NL335 product ion in MS2
PR2 = PR2.intensity; #intensity of the parent in MS2
allFAs = FA1.intensity; ; #intensity of the only fatty acid in MS2
```

```
QUERYNAME = pSMNL335; #search for propargylcholine-containing SMs upon click-reaction to N3Pal
DEFINE PR1 = 'C[51..89] H[99..199]  P[1] N5 O[8]' WITH DBR = (3.5,11), CHG = -1; #first precursor ion (click-reacted to N3Pal)
DEFINE PR2 = 'C[51..89] H[99..199]  P[1] N5 O[8]' WITH DBR = (3.5,11), CHG = -1; #second precursor ion (click-reacted to N3Pal)
DEFINE NL1 = 'C19 H33 N3 O[2]' WITH CHG = 0; #the characteristic neutral loss
```

```
IDENTIFY
PR1 IN MS1-
AND PR2 IN MS2-
AND NL1 IN MS2-

SUCHTHAT
isOdd(PR1.chemsc[C])
AND PR1.chemsc == PR2.chemsc

REPORT
NAME = "pSM(%d:%d)" % ((PR1.chemsc[C] - 41), (PR1.chemsc[db] - 6));
#the name of the unclicked lipid
FA1 = "(%d:%d)" %  ((PR1.chemsc[C] - 41), (PR1.chemsc[db] - 6)); #the
name of the only fatty acid
chemsc = PR1.chemsc; #the chemical sum formula of the lipid
C = "%d" %  (PR1.chemsc[C] - 41); #the number of double bonds in the
side chains excluding those of N3Pal
db = "%d" %  (PR1.chemsc[db] - 6); #the number of double bonds in the
side chains excluding those of N3Pal
MASS = "%4.4f" % "(PR1.mass)"; #the mass of PR1 with four digits
ERROR = "%2.2f ppm" % "(PR1.errppm)"; #the mass error in ppm
INTNL1 = NL1.intensity; #intensity of the characteristic NL335
product ion in MS2
PR2 = PR2.intensity; ; #intensity of the parent in MS2
```

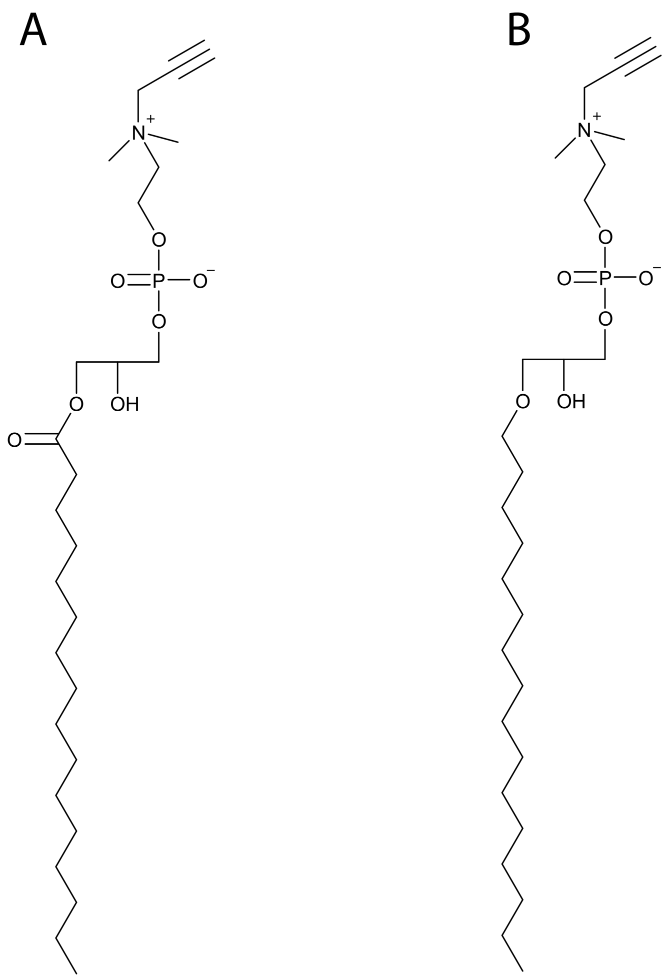

Figure S1. Synthetic lysolipids used as precursors in this study. The molecular structures of (A) palmitoyl-lyso-propargyl-PC (LpPC 16:0) and its ether analogue (B) 1-O-hexadecyl-2-lyso-sn-glycero-3-phosphopropargylcholine (LpPC O-16:0) are depicted.

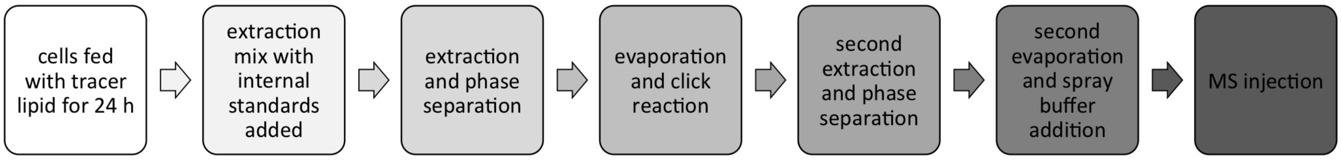

Figure S2. Diagram depicting the experimental flow. In this study a 24-well format with 45,000 cells was chosen. Sample volumes fit into 2 mL tubes. Sample preparation usually took 24 h with 15 min handling time.

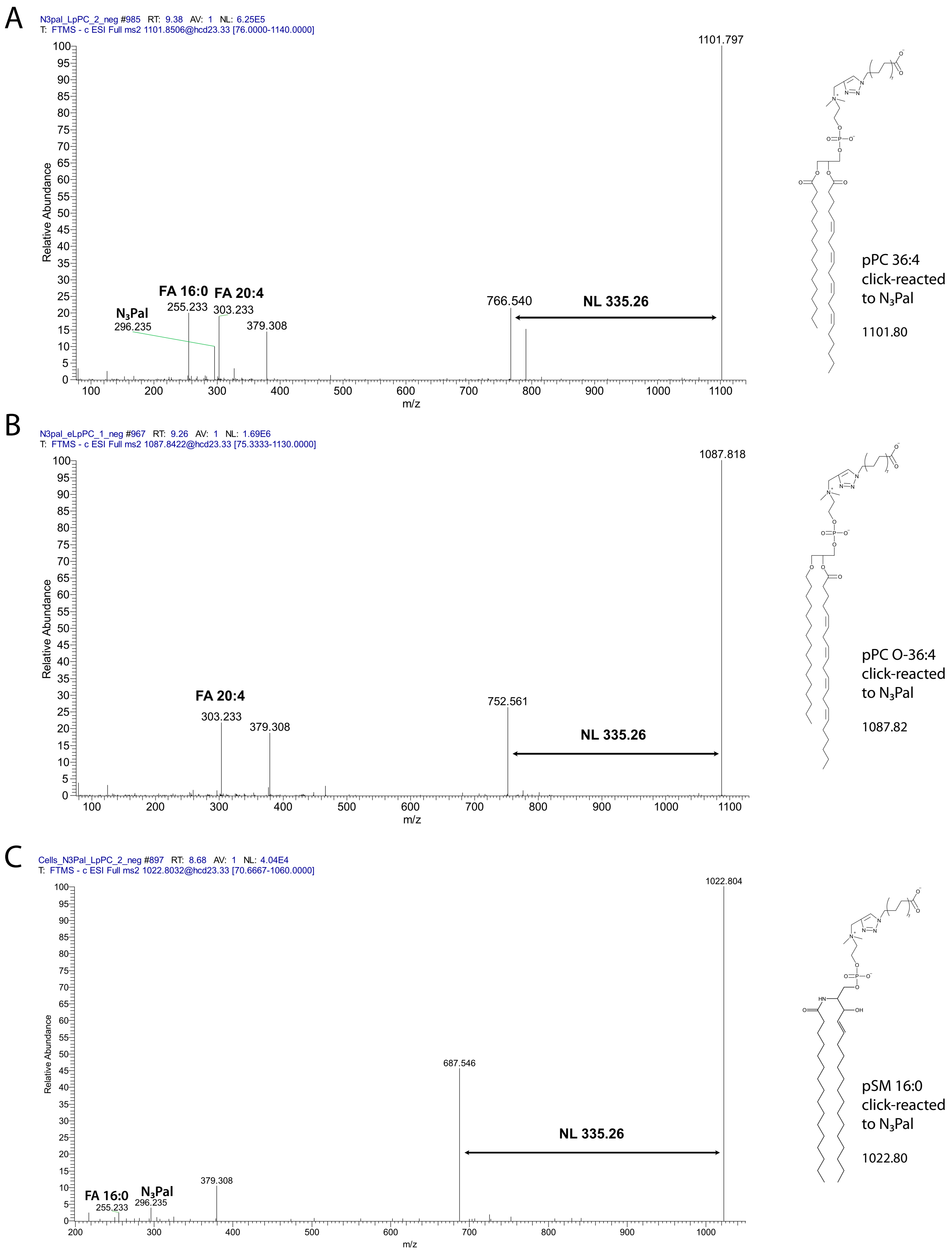

Figure S3. Analysis of propargylcholine phospholipids. Total lipids from bEND3 cells labeled with 20 µM LpPC 16:0 (A,C) or LpPC O-16:0 (B) for 24 h were isolated. Click-reaction with N_3Pal generated mass-shifted products, which were analyzed by negative mode electrospray ionization-tandem mass spectrometry. The MS2 fragmentation spectra of the cellular metabolites (A) pPC 36:4, (B) pPC O-36:4 and (C) pSM 16:0 are exemplarily shown on the left; their molecular structures on the right. For all labeled lipids the stereotypic NL 335.26 yielded a fragment that preserved lipid backbone information. Additionally, the fragments of the linked fatty acids can be detected allowing for side chain identification.

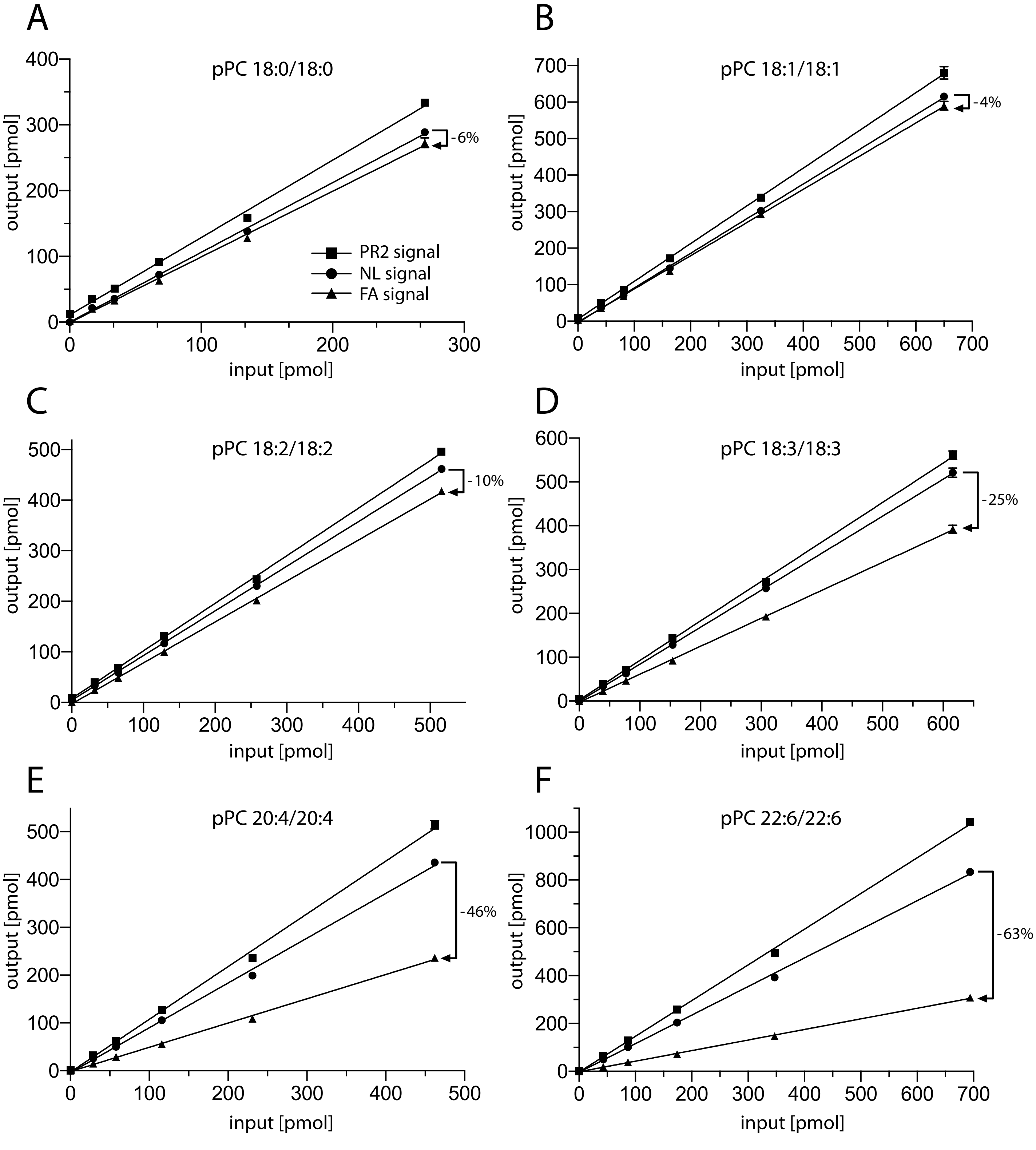

Figure S4. Analysis of the linearity, recovery, detection and quantification limits of the method. Total lipids isolated from 45,000 unlabeled bEND3 cells were mixed with increasing concentrations of synthetic (A) pPC 18:0/18:0, (B) pPC 18:1/18:1, (C) pPC 18:2/18:2, (D) pPC 18:3/18:3, (E) pPC 20:4/20:4 or (F) pPC 22:6/22:6 and (A-F) 240 pmol of pPC 31:1. Electrospray ionization-tandem mass spectrometry was performed. Samples were analyzed using the the N_3Pal reporter method and quantified using the pPC 31:1 internal standard. MS2 signals of the click-reacted lipid (PR2), its diagnostic fragmentation peak upon neutral loss of 335.26 (NL) and the sum of both fatty acids (FA) were used for calculations. Corrected analyte amounts detected upon all sample processing (output) are plotted against the amount of analyte added to the initial lipid mix (input). Data represent means ± SEM of five replicates. Linear regression of the curve corresponding to the NL signals yielded an estimate of the limit of detection (LOD; 1 pmol) and of the limit of quantification (LOQ; 4 pmol). Note the reduced slope of the curve corresponding to the FA signal in (D-F), indicating a underrepresentation of the PUFA signal.

Table S1. Analysis of non-targeted lipids of labeled cells.

	control (no supplement) pmol		LpPC 16:0 supplement pmol		LpPC O-16:0 supplement pmol	
Cer	143.0	±6.3	154.3	±10.8	166.3	±7.1
HexCer	48.4	±6.4	65.4	±3.8	121.9	±14.8
Hex2Cer	27.6	±8.1	21.9	±2.7	51.2	±11.8
PA	55.0	±11.4	133.6	±101.3	105.3	±33.3
PG	73.8	±3.5	60.4	±5.7	75.6	±6.6
PS	1805.9	±360.1	1407.7	±427.4	872.0	±207.6
MG	184.8	±68.4	181.2	±20.6	152.5	±16.3
DG	115.1	±11.3	143.7	±32.2	94.4	±16.8
TG	690.9	±205.2	876.5	±346.1	1168.3	±434.5
CE	1689.5	±263.1	1147.8	±207.2	1580.2	±154.9

Total lipids isolated from bEND3 cells labeled with 20 μM LpPC 16:0, LpPC O-16:0 or carrier for 24 h were quantified by electrospray ionization-tandem mass spectrometry. Sphingo-, glycerophospho- and neutral lipids were quantified using respective internal standards. Lipid amounts are shown as pmol per 45,000 cells and represent means ± SD, N=7. This data corresponds to the graphs depicted in Fig. 2A.

Table S2. Analysis of the (propargyl)choline-containing ether phospholipids.

	no supplement (endogenous PC O)		LpPC O-16:0 supplement		LpPC O-16:0 supplement	
	pmol		pmol by C171		pmol by N_3Pal	
PC O-/pPC O-30:0	21.2	±3.7	28.9	±7.5	41.0	±7.1
PC O-/pPC O-32:0	72.5	±10.5	36.3	±11.7	102.5	±12.3
PC O-/pPC O-32:1	76.0	±8.0	500.1	±92.5	770.8	±64.9
PC O-/pPC O-34:1	131.3	±23.3	903.7	±195.6	1568.6	±279.3
PC O-/pPC O-34:2	29.8	±5.7	106.8	±28.9	142.6	±31.5
PC O-/pPC O-36:1	10.7	±1.4	32.7	±12.9	44.0	±9.7
PC O-/pPC O-36:2	26.7	±4.6	7.5	±7.2	16.2	±4.1
PC O-/pPC O-36:3	18.5	±3.4	63.3	±17.7	34.2	±7.0
PC O-/pPC O-36:4	76.0	±14.2	274.0	±69.2	356.2	±83.5
PC O-/pPC O-36:5	15.8	±1.4	51.9	±13.9	52.7	±8.8
PC O-/pPC O-38:4	19.6	±2.3	18.1	±13.2	28.8	±5.8
PC O-/pPC O-38:5	72.8	±9.8	83.5	±24.5	129.0	±13.0
PC O-/pPC O-38:6	40.1	±6.2	200.1	±33.8	201.5	±41.7
sum	611	±67	2307	±391	3488	±505

Total lipids isolated from bEND3 cells labeled with 20 μM LpPC O-16:0 or carrier for 24 h were quantified by electrospray ionization-tandem mass spectrometry. The sum fatty acid composition of labeled pPC O species was analyzed by either the C171 or N_3Pal reporter method and quantified using the pPC 31:1 internal standard. The unlabeled (endogenous) PC O species were analyzed using the PC 31:1 internal standard. Each species is identified by two numbers: the first is the sum of radyl carbons, and the second is the sum of double bonds present in the two side chains. Lipid amounts are shown as pmol per 45,000 cells. Lipids less abundant than 25 pmol under all labeling conditions were omitted from table. Data represent means ± SD; N=7. This data corresponds to the graph depicted in Fig. 2C.
